# Minimally Invasive Parafascicular Surgery (MIPS) for Spontaneous Intracerebral Hemorrhage Compared to Medical Management: A Case Series Comparison for a Single Institution

**DOI:** 10.1155/2020/6503038

**Published:** 2020-06-13

**Authors:** Victoria L. Phillips, Anil K. Roy, Jonathan Ratcliff, Gustavo Pradilla

**Affiliations:** ^1^Rollins School of Public Health, Emory University, 1518 Clifton Road NE, Atlanta, GA 30322, USA; ^2^Department of Neurosurgery, Emory University School of Medicine, 1365 Clifton Road NE, Building B, Suite 2200, Atlanta, GA 30322, USA; ^3^Department of Emergency Medicine, Emory University School of Medicine, 1365 Clifton Road NE, Building B, Suite 2200, Atlanta, GA 30322, USA; ^4^Department of Neurology, Emory University School of Medicine, 49 Jesse Hill Jr Drive, SE, Suite 126, Atlanta, GA 30303, USA

## Abstract

**Objective:**

We compared the safety and effectiveness of minimally invasive parafascicular surgery (MIPS) as a frontline treatment for spontaneous supratentorial ICH to medical management. *Patients*. The sample consisted of 17 patients who underwent MIPS from January 2014 to December 2016 and a comparison group of 23 patients who were medically managed from June 2012 to December 2013. All had an International Classification of Disease (ICD) diagnosis of 431 and were treated at Grady Memorial Hospital, an urban, public, safety-net hospital.

**Methods:**

The primary endpoint was risk of inpatient mortality. Secondary endpoints were rates of inpatient infection and favorable discharge status, defined as discharge to home or rehabilitation facility. Demographics and pre- and postclinical outcomes were compared using *t*-tests, the Mann–Whitney test, and chi-squared tests for continuous, ordinal and categorical measures, respectively. Cox proportional hazard models were used to estimate the time to inpatient death. Logistic regression analyses were used to determine treatment effects on secondary outcomes. We also conducted exploratory subgroup analyses which compared MIPS to two medical management subgroups: those who had surgery during their hospitalization and those that did not.

**Results:**

Two patients (12%) died in the MIPS group compared to three (12%) in the medical management group. MIPS did not increase the risk of inpatient mortality relative to medical management. Rates of inpatient infection did not differ significantly between the two groups; eight MIPS patients (47%) and 13 medically managed patients (50%) contracted infections. MIPS significantly increased the likelihood of favorable discharge status (odds ratio (OR) 1.77; 95% CI, 1.12–21.9) compared to medical management. No outcome measures were significantly different between MIPS and the medical management subgroup without surgery, while rates of favorable discharge were higher among the MIPS patients compared to the medical management group with surgery.

**Conclusions:**

These data suggest that MIPS, as a frontline treatment for spontaneous ICH, versus medical management for spontaneous ICH warrants further investigation.

## 1. Introduction

Approximately 40,000 to 67,000 cases of spontaneous intracerebral hemorrhages (ICH) occur each year in the United States [1, 2]. The inpatient mortality rate for ICH from the US Nationwide Inpatient Sample was 26.4% [3]. Thirty-day mortality rates range from 35% to 52% with roughly half of deaths occurring within the first 24 hours of ictus; only 20% of survivors have full functional recovery at six months [4, 5].

Aggressive medical management is the standard of care for spontaneous ICH and consists of rapid diagnosis, coordination of care by a neurocritical team, and possible surgical interventions [6, 7]. Given the risk of expansion and damage to brain tissue, immediate surgical evacuation of hematomas is under investigation as a frontline treatment for ICH [8–10]. Two new surgical alternatives are: minimally invasive catheter-based hematoma evacuation and minimally invasive port-based surgical hematoma evacuation [11].

Randomized controlled trials (RCT) using minimally invasive, image-guided, catheter-based aspiration and treatment with rtPA for basal ganglia ICH have proven safe compared to both conventional craniotomy (MISPTT) and medical management (MISTIE I, II, and III-on-going) [12–16].

Less is known about minimally invasive parafascicular surgery (MIPS) with a transulcal access corridor. The approach has five core components: image interpretation to determine surgical corridors that are less disruptive to white matter tracts; intraoperative navigation that constrains the surgical trajectory to the preplanned corridor; access with a minimally invasive port that enables transulcal cannulation and decreases white matter shearing; visualization of the surgical field using an exoscopic optical system; and clot evacuation utilizing a mechanical suction and resection tool specifically designed for transportal surgery. The technique utilized has been described previously by our group and others [17, 18].

A recent patient case series provides evidence on the effectiveness and safety of MIPS. Reporting on 39 patients from eleven centers, Labib et al. [16] achieved a clot evacuation of ≥90% in 72% of patients. In a second series of 18 patients from a single center, Bauer et al. [18] achieved a mean clot evacuation of 96% and a median GCS improvement from admission to discharge.

MIPS has also been shown to be associated with extremely low inpatient mortality rates with two studies reporting rates of inpatient mortality rates of 0%, while another, including a cerebellar stroke, reported an ICH-related mortality rate of 36% [16, 18, 19].

Sujijantarat et al. [20] conducted the single study comparing MIPS and medical management and found significantly lower inpatient mortality rates among the MIPS group (6.25% versus 75%). However, while the MIPS and medical management groups were matched on stroke location, volume, and GCS score, they were not matched on comorbidities, and physicians and families determined allocations of patients to treatment groups undermining comparability between the two groups.

The fact that 75% of deaths in the medical group resulted from withdrawal of care suggests a difference in acuity between the two groups.

**Figure 1:**
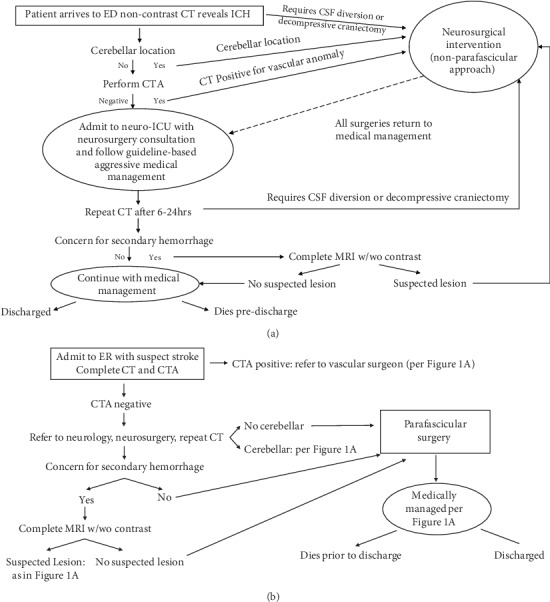
(a) Flow diagram of medical management of intracerebral hemorrhage (ICH) stroke patients: 2012-2013. (b) Flow diagram of MIPS.

Grady Memorial Hospital admits approximately 640 strokes annually, of which an estimated 21% are ICH. It began using MIPS in 2014 to inform development of a clinical protocol for the procedure.  Figure 1(a) shows the clinical pathway for ICH patients from June 2012 to December 2013, prior to the introduction of MIPs. Patients in need of cerebrospinal fluid (CSF) diversion, a decompressive craniectomy, who had a cerebellar stroke or a vascular anomaly were referred for immediate surgery. The remainder were medically managed with subsequent results from computed tomography (CT) scans or magnetic resonance images (MRI) possibly leading to later surgery. All continued medical management postsurgery, and either died inpatient or were discharged to the community.

The introduction of MIPS, shown in Figure 1(b), changed this pathway whereby a new group of patients, namely those without a cerebellar stroke, with no suspected secondary hemorrhage or lesions were now referred for immediate surgery. Intubation for either group, MIPS or medical management, occurred at Emergency Room admission, and intubation post procedures or use of a vent depended on the clinical condition of the patient with the same protocols used for each group.

In preparation for a randomized control trial (RCT), we compared data on ICH patients who received MIPS during the trial period, January of 2014–December of 2016, with that for matched patients who had received care in the prior (pre-MIPS) period, June of 2012–December of 2013. We used this approach as patients were not randomized to MIPS during the protocol development period and all patients referred to surgery underwent surgery; thus no similar, contemporaneous comparison group was available.

Our primary endpoint was the risk of inpatient mortality between the groups, and the secondary endpoints were rates of inpatient infection and favorable discharge status. Favorable discharge status was defined as discharge to home or rehabilitation setting and unfavorable as discharge to a long-term care facility (LTC), a long-term care acute facility (LTAC) or hospice or death. Percent clot evaluation could not be compared between the two groups as post-volume measures were not available for the medically managed cohort as scans prior to discharge were not part of medical management guidelines at the time.

We also compared MIPS patients with (1) medically managed patients who ultimately underwent any form surgery and (2) medically managed patients who did not undergo surgery.

The study was funded through an unrestricted grant from the NICO Corporation, Indianapolis, Indiana. The BrainPath port (NICO Corporation, Indianapolis, Indiana) and the NICO Myriad (NICO Corporation, Indianapolis, Indiana) aspirator were used in all surgeries. The funder had no role in study design, data collection, data analysis, data interpretation, writing of the report, or decision to pursue publication.

The study was approved by the hospital's Institutional Review Board. It involved analysis of secondary data only. MIPS patients were those participating in the patient registry for Minimally Invasive Subcortical Parafascicular Access for Clot Evacuation (Mi SPACE) [21]. Identifiers for the groups were removed prior to analysis. Data for the comparison group were provided in deidentified form. Given the data are drawn from a single hospital with a relatively small sample, the dataset will not be made available due to patient privacy concerns. However, specific data questions may be directed to the corresponding author who will address them, as possible, on a case by case basis.

## 2. Methods

All patients were treated at Grady Memorial Hospital, a single, urban, public, safety-net hospital with a comprehensive stroke center. We compared data on ICH patients who received MIPS during the trial period, January 2014–December 2016, with that for matched patients who had been medically managed in the prior period, July 2012–December 2013. We used this approach as during the protocol development period for MIPS, all patients referred to surgery received surgery, and no unbiased contemporaneous comparison group was available.

MIPS patients were identified from the center's ICH Mi SPACE registry [21]. Inclusion criteria were ICD 431 diagnosis, ICH volume ≥ 20 ml and ≤ 80 ml, and having a lobar, basal ganglia, or thalamic ICH. Infratentorial hemorrhages and patients with underlying tumor or vascular lesions were excluded. Also excluded were patients with diabetes, dementia, AIDS, documented alcohol or drug dependence, cancer and liver or kidney disease, and those with surgeries performed >24 hours of admission. One surgeon performed all MIPS surgeries.

The medical management cohort was comprised of all ICH patients admitted to the hospital during the period June 2012 to December 2013 who met the inclusion criteria. Inclusion/exclusion criteria for the group were identical to those for the MIPs patients. Medically managed patients who underwent any kind of surgery were retained in the sample; none were treated using BrainPath (NICO Corporation, Indianapolis, Indiana) as it was not available during this time period. Two authors (AKR and GP) reviewed all files to confirm eligibility.

Demographic, clinical, and outcome data were collected from the Mi SPACE registry, patient clinical records, including physician and nurse notes, and patient billing records. The primary endpoint was risk of inpatient mortality, while secondary endpoints were rates of infection and favorable discharge status.

Patient characteristics and pre- and postclinical outcomes were compared using *t*-tests, Mann–Whitney tests if the distribution was nonnormal and chi-squared tests for continuous, ordinal and categorical measures, respectively. Statistical significance is *p* ≤ 0.05. Means and standard deviations (SD) were reported for continuous measures unless evidence of skewness was present, whereby medians and interquartile ranges (IQR) were reported [22].

A Cox proportional hazard model was used to estimate the time to inpatient death. Given the sample was not randomized, logistic regression analyses with bootstrapped confidence intervals were used to determine the treatment effects on infection and favorable discharge status. Model covariates, limited to reflect the sample size, were the Intracerebral Hemorrhage (ICH) scale score; whether the patient had a lobar stroke; whether the patient was placed on a ventilator during the hospitalization, or whether the patient had an infection [23]. These measures were used to capture severity of stroke, position, and inpatient events likely to affect outcomes. Statistical analyses were conducted using the IBM SPSS (version 24) software (SPSS Inc., Chicago, Illinois) and SAS (9.4 MG) software (SAS Institute Inc., Cary, NC, USA) [24, 25].

We also conducted two exploratory analyses. We divided the medically managed patients into two subgroups: (1) those who underwent any surgery during their inpatient stay and (2) those who did not. We compared each subgroup, proxying more and less complicated medically managed patients, respectively, to MIPS patients.

## 3. Results

### 3.1. Demographics and Baseline Clinical Characteristics

Our institution admits approximately 640 strokes annually, of which 21% (268) are ICH. During the two-year MIPS trial period, 52 patients underwent surgery, of which 17 met study the inclusion criteria. Reasons for exclusion in the treatment group were patient comorbidities, 55%; outside the volume range, 38%; and cerebellar strokes, 7%. In the medically managed group, 130 ICH patients were screened with 23 out of 130 cases meeting study inclusion criteria. Reasons for exclusion were patient comorbidities, 46%; outside the volume range, 43%; and cerebellar strokes, 11%.

Table 1 provides data on demographic and the preclinical values for the full sample and the two treatment groups. No patients had missing data on the study variables given that investigators had access to clinical notes and medical and billing records. Comparisons showed no significant differences between the MIPS and medically managed groups on demographic and preclinical measures. Mean age for the sample was 62.6 (±14.5); 49% were male; and 70% were black.

For the full sample, the median GCS Admission (GCSADM) score was 12 (IQR 9, 13), while the median modified Rankin Scale (mRS) score on admission was 4 (IQR 3, 5). The median ICH volume was 36.3 (IQR 28, 50), and 44% of the sample had a lobar ICH with the majority of hemorrhages located on the left side; 34.9% of the sample presented with an intraventricular hemorrhage (IVH).

### 3.2. Patient Outcomes

Data on outcomes for the full sample and by treatment group are shown in Tables 2 and 3. For the full sample, 72% of patients were placed on a ventilator during their stay. The median mRS score at discharge was 5 (IQR 4, 5), while that for GCS was 12 (IQR 8, 12). GCS scores from admission to discharge declined for 42% of patients in the medical group, compared to 29% in the surgical group. Similar percentages of patients in both groups had no change, while 41% in the surgical group compared to 31% in the medical arm showed GCS score improvement, but the difference was not statistically significant.

Seventy-two percent clot evacuation was achieved in the MIPS group; data were not available for the medically managed group. Based on descriptive statistics, no values differed significantly between the treatment groups; more favorable discharges destinations for the BrainPath (Nico Corporation, Indianapolis, Indiana) group trended higher at *p* = 0.080 relative to medical management.

**Figure 2:**
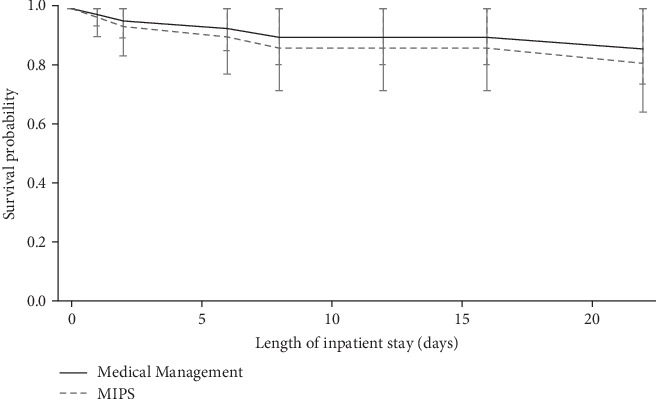
Adjusted survival from admission by the treatment group. Bars represent 95% confidence intervals.

Three (12%) patients died, on days 1, 2, and 8, in the medical group compared to two (12%), on days 6 and 22, in the MIPS group. As shown in Figure 2, undergoing MIPS had no protective effect on survival as the confidence intervals for the curves overlap. Having a higher ICH scale score increased the likelihood of inpatient mortality, shown in Table 3, (odds ratio (OR) 4.14; 95% CI, 1.06 - 16.19).

Thirteen (50%) patients in the medically managed group and 8 (47%) in the MIPS surgery group developed infections. One infection was surgically related. Pulmonary and urinary tract infections comprised the vast majority of infections in the sample at 56% and 28%, respectively.

Regression results for analysis of the secondary endpoints are shown in (Table S1). Having MIPS surgery compared to medical management had no effect on the odds of developing an inpatient infection.

Regarding discharge status, nine (35%) in the medical group and nine (53%) in the surgery group had favorable discharge destinations, defined as discharge to home or to a rehabilitation facility. Having surgery increased the likelihood of favorable discharge status (odds ratio (OR) 1.77; 95% CI, 1.12–21.9), while having been placed on a vent decreased them (odds ratio (OR) -3.34; 95% CI, -41.32- -1.75).

### 3.3. Exploratory Subgroup Analyses

We divided the medically managed patients into two subgroups: (1) those who underwent any surgery during their inpatient stay (*n* = 16) and (2) those that did not (*n* = 10). We compared each subgroup, proxying more and less complicated medically managed, respectively, to MIPS patients.

Sixteen (62%) patients in the medically managed group went on to have some form of surgery during their inpatient stay. In the medically managed group, there were: 2 decompressive craniotomies, 4 angioplasties, 5 diagnostic angiographies, 4 catheter aspiration of the nasotrachia, and 1 incision of the larynx. In the treatment group there were: 1 decompressive craniotomy, 3 diagnostic angiographies, 1 bronchoscopy, and 1 tracheostomy.

Table 4 compares MIPS patients with the medical subgroup who underwent any surgery. No patient characteristics were significantly different. The MIPS group had significantly higher rates of favorable discharge, 43% compared to 25% (*p* = 0.03), compared to the medical subgroup with a subsequent surgery.

Table 5 shows the results of comparisons between MIPS patients and medically managed patients that did not undergo surgery. No significant differences existed between the two subgroups. Improvement in GCS scores from admission to discharge were 41% to 30% for BrainPath (NICO Corporation, Indianapolis, Indiana) compared to medical management, while respective rates of favorable discharge, 53% and 50%, were similar.

## 4. Discussion

Minimizing contemporaneous, nonrandom selection issues, we used two natural patient case series from consecutive points in time to examine the safety and effectiveness of MIPS for spontaneous supratentorial ICH relative to medical management from admission to discharge.

We present a comparison of two patient cohorts: one treated with MIPs during January 2014 to December 2016 and one treated with medical management from June 2012 to December 2013. The results show that MIPS is safe relative to medical management as neither the rates of inpatient mortality nor infection differs between the two groups. MIPS shows potentially improved outcomes relative to medical management in terms of discharge status.

Our exploratory analyses compared MIPS and medical management based on whether medically managed patients underwent any surgery during their hospitalization. Safety endpoints did not differ between MIPS patients and either subgroup, while MIPS increased the odds of favorable discharge status when compared to medically managed patients who underwent any form of surgery. While exploratory, these results suggest that MIPS performs as well as medical management for less complicated cases and potentially better in terms of outcomes for more complicated cases.

### 4.1. Comparisons to Prior Studies

Our results, particularly related to mortality, differ substantially from prior cases series. Our rate for the MIPS group was higher than that of previous studies (12% versus 0%), and unlike Sujijantarat et al. [20], we found no survival advantage in using MIPS relative to medical management.

Lower rates from earlier work likely reflect differences in patient samples. Labib et al. [16] reported on cases from 12 recently trained surgeons. In Sujijantarat et al. [20], the comparison groups were contemporaneous but not randomly assigned, and it is not clear from what larger patient pool the medically managed group was drawn.

Our goal was to minimize nonrandom, contemporaneous selection issues; however, for the MIPS group, some form of institutional selection may be present. The treatment was introduced at the center in January of 2014, the beginning of the observation period for the MIPS cohort. The neurocritical care unit was responsible for patient referrals for surgery. It may have initially referred patients for whom medical management was viewed as less likely to be effective as opposed to positive surgical selection. Should this hold, the MIPS inpatient mortality rate may be overestimated. Whether or to what degree this bias exists is beyond the scope of the current study.

Our higher inpatient mortality rate may also reflect the greater heterogeneity of our patient population. The sample in Labib [16] included patients with minimal or no IVH compared to 35% of our sample. We also had a larger percentage of lobar strokes than Sujijantarat [20] (44% versus 31%). Neither paper provides information on patient comorbidities which we have found to significantly impact patient outcomes.

Our study is not directly comparable to larger multicenter randomized controlled trials. Our patient population was treated under the MIPS protocol described. The group also suffered more severe hemorrhages than those in many RCT. We did not require patients to have an expectation of survival for 180 days (INVEST) [31]. We also did not exclude patients having or anticipating the need for decompressive craniectomy (INVEST), having IVH (STICH II), or having a thalamic stroke (MISTIE III) [10, 15, 26].

We used favorable discharge status, either to home or to a rehabilitation facility, as a proxy for clinical benefit. We viewed these as favorable discharge destinations as both groups are expected to improve as a result of therapy. With home placement, patients receive in-home or outpatient therapy. Those transferred to a rehabilitation facility must be able to tolerate physical therapy and are expected to be discharged home or to an assisted living facility.

However, discharge destination can be influenced by bed availability, patient preference, location of a facility, and insurance, effects we could not measure. We do not know how well our proxy measure correlates with other traditional measures, evaluated over a longer time period, such as the 30-day mRS. Studies of ischemic stroke show that discharge destination is linked with positive 90-day outcomes [27–29].

Patients who were treated through medical management may not be comparable to those patients treated contemporaneously with MIPS. Medical management may also have improved over the period. If so, our results may overestimate differential outcomes between MIPS and medical management. Alternatively, surgical skill may also improve over time which may underestimate any relative treatment benefits of MIPS.

### 4.2. Limitations

Our study sample is small, and our models were limited due to sample size. The size of the regression coefficients should be viewed with caution as coefficients on larger samples are likely to be smaller. The subgroup findings must be viewed as exploratory given the small sample sizes.

Our study was conducted at a single site which limits it generalizability. Our patient population in terms of underlying health status may differ from that of other facilities. However, our study has high internal validity as it controls for surgical skill and patterns of medical management.

Multicenter sites raise concerns about variation as patients are randomized across not within sites. Careful training can offset surgical variation, but not likely medical management. Many centers have well established pathways for acute ischemic strokes; few, however, have similar protocols for ICH although two have been developed [4, 11, 30]. Facility variations have been shown to significantly affect patient mortality even after adjustment for other factors, including stroke [31].

## 5. Conclusion

Current AHA guidelines report that surgery has not been shown to have improved outcomes over neurocritical team management for supratentorial ICHs. Our results demonstrate the safety profile of MIPS relative to medical management and its potential clinical benefits using two patient case series. Our findings suggest that MIPS warrants further investigation and a RCT (ENRICH) is now underway to investigate its performance relative to medical management [32].

## Figures and Tables

**Table 1:** Demographic and preclinical data for the full sample and by treatment group: medical management versus minimally invasive parafascicular surgery (MIPS).

Variables	Full sample (*n* = 43)	Medical management (*n* = 26)	MIPS (*n* = 17)	*p* value
Patient demographics				
Age years (SD)	63 (14.5)	64 (14.6)	61 (14.7)	0.53
Male, *n* (%)	21 (49)	13 (50)	8 (47)	0.85
Black, *n* (%)	30 (70)	21 (81)	9 (53)	0.11
Preclinical characteristics				
Diabetes, *n* (%)	6 (14)	3 (12)	3 (18)	0.67
Modified Rankin scale (mRS) score on admission median (IQR)	4 (3, 5)	4 (3, 5)	4 (3.5, 5)	0.62
Glascow coma scale score (GCS) on admission median (IQR)	12 (9, 13)	11.5 (9, 13.2)	11.3 (9.5, 13.5)	0.95
Acute Physiology and Chronic Health Evaluation II (APACHE II) mean (SD)	11.2 (4.6)	10.9 (4.6)	11.7 (4.8)	0.58
Intracerebral Hemorrhage Scale score median (IQR)	2 (1, 2)	2 (1, 3)	2 (1, 2)	0.42
Prevolume, ml median (IQR)	36.3 (28, 50)	36.3 (26, 49)	36.4 (29, 58)	0.87
Stroke type				0.40
Basal ganglia, *n* (%)	21 (49)	13 (50)	8 (47)	
Lobar stroke, *n* (%)	19 (44)	10 (38)	9 (53)	
Thalamic, *n* (%)	3 (7)	3 (12)	0	
Location				0.94
Left, *n* (%)	23 (54)	14 (54)	9 (53)	
Right, *n* (%)	20 (40)	12 (46)	8 (33)	
Intraventricular hemorrhage, *n* (%)	15 (35)	9 (35)	6 (35)	0.96

**Table 2:** Outcome data for the full sample and by treatment group: medical management versus minimally invasive parafascicular surgery (MIPS).

Variables	Full sample (*n* = 43)	Medical management (*n* = 26)	MIPS (*n* = 17)	*p* value
Patient outcomes				
Vent, *n* (%)	31 (72)	17 (65)	14 (82)	0.31
Inpatient infection, *n* (%)	21 (49)	13 (50)	8 (47)	0.85
Deaths, *n* (%)	5 (12)	3 (12)	2 (12)	0.98
Modified Rankin scale (mRS) at discharge median (IQR)	5 (4, 5)	5 (3.75, 5)	4 (3, 5)	0.74
Glascow coma scale score at discharge median (IQR)	12 (8, 12)	10.5 (7.8, 15)	13 (9, 14)	0.75
Discharge status				0.08
Home, *n* (%)	6 (14)	5 (19)	1 (6)	
Rehabilitation facility, *n* (%)	12 (28)	4 (15)	8 (47)	
Long-term care skilled nursing facility, *n* (%)	8 (19)	7 (27)	1 (6)	
Long-term care acute hospital, *n* (%)	11 (26)	6 (23)	5 (29)	
Death or hospice, *n* (%)	6 (14)	4 (15)	2 (12)	
Glascow coma scale improvement, *n* (%)	15 (39)	8 (35)	7 (47)	0.464

**Table 3:** The Cox proportional hazard model for inpatient death.

Variable	Hazard ratio	95% CI	*p* value
Treatment group (1 = MIPS)	1.52	(0.21-10.96)	0.68
Having a lobar stroke	1.01	(0.14-7.21)	0.99
Intracerebral hemorrhage scale score	4.14	(1.06-16.19)	0.041

Being on a vent and dying were perfectly correlated, so being on a vent could not be included in the regression.

**Table 4:** Demographic, preclinical, and outcome data by minimally invasive parafascicular surgery (MIPS) and medical surgery.

Variables	Full sample (*n* = 33)	MIPS (*n* = 17)	Medical surgery (*n* = 16)	*p* value
Patient demographics				
Age years (SD)	61 (14.2)	61 (14.7)	61 (14.2)	0.97
Modified Rankin scale score on admission (median)	4 (3, 5)	4 (3.5, 5)	3.5 (3, 4)	0.30
Glasgow coma scale score on admission median (IQR)	12 (10, 14)	11.3 (9.5, 13.5)	12 (10, 14.75)	0.51
Intracerebral hemorrhage scale score median (IQR)	2 (1, 2)	2 (1, 2)	2 (1, 2)	0.95
Prevolume, ml median (IQR)	36.5 (29, 58)	41.8 (29, 58)	37.7 (27.7, 57.4)	0.97
Stroke type				0.20
Basal ganglia, *n* (%)	17 (52)	8 (47)	9 (56)	
Lobar stroke, *n* (%)	14 (42)	9 (53)	5 (31)	
Thalamic	2 (6)	0 (0)	2 (12)	
Patient outcomes				
Inpatient infection, *n* (%)	16 (43)	8 (47)	8 (33)	0.87
Deaths, *n* (%)	3 (9)	2 (12)	1 (0)	0.58
Modified Rankin scale at discharge median (IQR)	5 (4, 5)	4 (4, 5)	5 (3.75, 5)	0.96
Glascow coma scale score at discharge median (IQR)	13 (8.5, 14)	13 (9, 14)	12.5 (8.25, (14.75)	0.87
Discharge status				0.03
Home, *n* (%)	4 (4)	1 (6)	3 (19)	
Acute rehabilitation facility, *n* (%)	9 (27)	8 (47)	1 (6)	
Skilled nursing facility, *n* (%)	7 (21)	1 (6)	6 (37)	
Long-term acute hospital, *n* (%)	10 (30)	5 (29)	5 (31)	
Died or hospice, *n* (%)	3 (9)	2 (12)	1 (6)	
Glascow coma scale score improvement, *n* (%)	12 (36)	7 (41)	5 (31)	0.55

**Table 5:** Demographic, preclinical, and outcome data by Mi SPACE and medically managed patients without surgery.

Variables	Full sample (*n* = 27)	Mi SPACE (*n* = 17)	No medical surgery (*n* = 10)	*p* value
Patient demographics				
Age years(SD)	63.8 (14.81)	60.9 (14.68)	68.7 (14.46)	0.192
Modified Rankin scale score on admission median (IQR)	4 (3, 5)	4 (3.5, 5)	4.0 (2.75, 5)	0.706
Glascow coma scale score on admission median (IQR)	11 (9, 13)	11.3 (9.5, 13.5)	10.5 (6.25, 13)	0.695
Intracerebral hemorrhage scale score median (IQR)	2 (1, 2)	2 (1, 2)	2 (1, 3.5)	0.897
Prevolume, ml median (IQR)	34 (28, 50)	41.8 (29, 58)	33.6 (25.75, 49)	0.695
Stroke type				0.6404
Basil ganglia, *n* (%)	14 (52.0%)	9 (47.06%)	5 (50.0%)	0.410
Lobar stroke, *n* (%)	12 (44.4%)	8 (52.94%)	4 (40.0%)	
Thalamic, *n* (%)	1 (3.7%)	—	1 (10%)	
Patient outcomes				
Inpatient infection, *n* (%)	10 (43.48%)	8 (47.06%)	5 (33.33%)	0.883
Deaths, *n* (%)	2 (8.70%)	2 (11.76%)	2 (0%)	0.561
Modified Rankin scale at discharge median (IQR)	4 (4, 5)	4 (4, 5)	4.5 (3.75, 5.25)	1.000
Glascow coma scale score at discharge median (IQR)	12 (12, 14)	13 (9, 14)	9 (5.25, 15)	0.695
Discharge status				0.417
Home	3 (11.1)	1 (5.9)	2 (20.05)	
Acute rehabilitation, *n* (%)	11 (40.7%)	8 (47.0)	3 (30.0%)	
Long-term skilled nursing facility, *n* (%)	2 (18.5)	1 (5.9)	1 (10.0%))	
Long-term acute care hospital, *n* (%)	6 (22.0%)	5 (29.4)	1 (10.0%)	
Died/hospice, *n* (%)	3 (13.0%)	2 (11.8%)	1 (10.0%)	
GCS improvement	10 (37%)	7 (41.2%)	3 (30%)	0.561

## Data Availability

Given the data are drawn from a single center with a relatively small sample, the dataset will not be made available due to patient privacy concerns. However, specific data questions may be directed to the corresponding author who will address them, as possible, on a case by case basis.

## Abbreviations

CSF: Cerebrospinal fluid
CT: Computed tomography
CTA: Computed tomography angiography
ER: Emergency room
GCS: Glascow Coma scale
ICH: Intracerebral cerebral hemorrhage
ICHSC: Intracerebral hemorrhage scale score
ICD: International Classification of Diseases
IQR: Interquartile range
IVH: Intraventricular hemorrhage
LTC: Long-term nursing home care
LTAC: Long-term acute care hospital
MIPS: Minimally invasive parafascicular surgery
MISTIE: Minimally invasive endoscopic surgery vs. medical management in supratentorial intraparenchymal hemorrhage
Mi SPACE: Minimally invasive subcortical parafascicular access for clot evacuation
mRS: Modified Rankin score
RCT: Randomized controlled trials
SD: Standard deviation
SNF: Skilled nursing facility
STICH: Surgical trial in intracerebral hemorrhage.

## Supplementary Materials

Supplementary Materials Table S1: logistic regression results for inpatient infection and favorable discharge status.
