# Supplementary material for: Minimally Invasive Parafascicular Surgery (MIPS) for Spontaneous Intracerebral Hemorrhage Compared to Medical Management: A Case Series Comparison for a Single Institution
Source: Stroke Res Treat. 2020 Jun 13;2020:6503038. doi: 10.1155/2020/6503038 (PMC7306855; doi:10.1155/2020/6503038)
Supplement: Supplementary Materials — Table S1: logistic regression results for inpatient infection and favorable discharge status. [file 6503038.f1.pdf]

Supplement 1: Logistic Regression Results for Inpatient Infection and Favorable Discharge Status

Supplement Table 1: Logistic Regression Results for Inpatient Infection

Variable	Odds Ratio	95% CI	P-value
Treatment Group (1 = MIPS)	1.25	(0.35 - 4.46)	0.729
Having a Lobar Stroke	0.98	(0.29 - 3.38)	0.977
Intracerebral Hemorrhage Scare Score	1.12	(0.55 - 2.26)	0.757
Having been on a ventilator	0.62	(0.15 - 2.55)	0.510

Supplement Table 2: Logistic Regression for Favorable Discharge Status

Variable	Odds Ratio	95% CI	P-value
Treatment Group (1 = MIPS)	1.77	(0.168 - 21.992)	0.019
Having a Lobar Stroke	-0.59	(-2.339 - 0.895)	0.497
Intracerebral Hemorrhage Scare Score	-0.43	(-19.328 - 1.966)	0.379
Having been on a ventilator	-3.34	(-41.318 - -1.754)	0.001
